# Dental Notes

**Published:** 1899-05

**Authors:** F. S. Casper

**Affiliations:** Austin, Texas


For Texas Medical Journal.

### Dental Notes.

BY F. S. CASPER, D. D. S., AUSTIN, TEXAS.

MARYLAND LAW IN QUESTION.—An unregistered dentist, who has been indicted by the State Dental Board, will endeavor to prove that the law is unconstitutional, because, he says, it empowers the Board to pass upon the qualifications of those who desire to practice dentistry, yet does not state what the qualifications shall be.—*Dental Digest.*

SMOKERS' TEETH.—It has been found that the teeth of smokers are less liable to decay than those of non-smokers, and it has been found by scientific research that *leptothrix buccalis* and the other germs found in the mouth are rendered innocuous by tobacco smoke, and it is an established fact that it also entirely destroys, or retards, the development of the bacillus of cholera, of anthrax, and of pneumonia.—*Weekly Dentist.*

[And it also destroys the vital energy of man, and a healthy breath.—ED.]

TREATMENT.—Until a better acquaintance with the etiology of this disease is obtained, no definite line of treatment can be outlined. The teeth should be thoroughly cleansed of all deposits; with suitable scalers all calcarious salts should be scraped from under the gums, wash out the pocket with dilute per oxide of hydrogen, then take a small rope of cotton, saturated with 25 per cent. hydrochloric acid, carry this under the gum, speedily remove, and counteract the effect of the acid with a rope of cotton dipped into a solution of soda bicarbonate, wash out with warm water, dry the parts, make a paste of iodoform, glycerine and quinia sulphate, with a blunt instrument pack this paste under the gums, and discharge patient with the admonition to return in three days, when the pocket should again be syringed with antiseptic and the paste removed. If the teeth are loose, they should be wired together until they become firm. This treatment, while it is not infallible, gives good satisfaction.

We note (from *The Dental Digest,* April, 1899) that Dr. Sylvanus Davis, D. D. S., of Denver, Colorado, gave a very interesting clinic before the Denver Dental Society January 12, 1899, in which he demonstrated to the satisfaction of those present that gold foil can be made to cohere under water. The doctor filled two teeth with the cavities submerged, leaving the surface of the fillings perfectly smooth and free from pits, which is difficult to accomplish by the dry method, with the rubber dam.

We do not doubt that Dr. Davis accomplished all he claimed, viz.: that submerged gold can be made adhesive. It has been claimed by the best operators in the days of "soft or non-cohesive gold workers" that teeth could be successfully filled under moisture; doubtless many teeth were saved in this manner for a long time, the duration depending upon the condition of the fluids of the mouth at the time the operation was performed. We are compelled to admit the ideas of such men as Professors Black and Miller. The latter, a noted bacteriologist of Berlin, in his great work says: "The active cause of caries of the teeth is an acid, and micro-organisms are the active agents in forming acids." Seventeen distinct varieties of microbes have been found in the human saliva to date. It would therefore appear, that although the theories advanced by Dr. Davis are an accomplished fact, very little benefit can be derived unless we could substitute sterilized water for the saliva. Dr. Davis deserves great credit for his researches in this direction, and we hope to hear more from him.

---

PYORRHEA ALVEOLARIS.—This disease, as its name implies, is generally detected by a flow of lymph or pus from a pocket under the gum around one or more teeth affected, although cases no doubt exist where there seems to be a total absence of an opening into a pocket, simply a wasting away of the process under the gum, leaving a line of concretion over the margin of the gum. As stated above, one or more teeth may be attacked, the alveolar process wasting away, until the tooth becomes loose and falls out of the socket, before the adjoining member becomes affected.

Twenty-five years ago, this troublesome disease was little known; today it is alarmingly on the increase.

As to the causation of pyorrhea alveolaris, little is known. Many theories have been advanced as to its origin and treatment; all seem to fail, however, after mature investigation. One of the causes assigned is, an excess of lime salts in the system, thereby causing a deposit of calcarious salt on the tooth, which gradually forms against the free gum margin, causing irritation, inflammation, and finally suppuration, when the pocket is formed; and the deposit continues under the gum, until the alveolar process is involved and wastes away. Another, and by far the most plausible cause assigned, is "lithemia." We incline to this opinion, as we have undisputable evidence of the existence of an excess of uric acid in every case we have treated, and we venture the assertion that the obstinacy of the disease depends upon the amount of uric acid contained in the system. We are not prepared to say that a deposit of calcarious salts in the kidneys and other organs, may not be the primary cause of lithemia, but we do believe that if the cause was strictly attributable to the lime salts, it would yield more readily to treatment. On the other hand, if the cause is from an excess of uric acid, it is clear that the tissues are wasting away, and we cannot expect healthy granulations to spring up when the general tendency of the system is to break down healthy tissues. One writer, Dr. O. N. Heise, M. D., D. D. S., of Cincinnati, says: "To such an extent has this disease become prevalent that at least 25 per cent. (according to Dr. Talbot) of all people over 25 years of age are thus more or less afflicted." Here again we have evidence of the theory of "lithemia," for it is seldom that those under 25 years of age are subject to lithemia.

